# *Bletilla striata* Polysaccharide Promotes Diabetic Wound Healing Through Inhibition of the NLRP3 Inflammasome

**DOI:** 10.3389/fphar.2021.659215

**Published:** 2021-04-26

**Authors:** Yan Zhao, Qibin Wang, Shan Yan, Jun Zhou, Liangyong Huang, Haitao Zhu, Fang Ye, Yonghong Zhang, Lin Chen, Li Chen, Tao Zheng

**Affiliations:** ^1^ Institute of Wudang Traditional Chinese Medicine, Taihe Hospital, Hubei University of Medicine, Shiyan, China; ^2^ College of Pharmacy, Hubei University of Medicine, Shiyan, China; ^3^ Department of Pharmacy, Taihe Hospital, Hubei University of Medicine, Shiyan, China

**Keywords:** *Bletilla striata*, polysaccharide, diabetic foot ulcer, NLRP3 inflammasome, high glucose

## Abstract

The aim of this study was to evaluate the therapeutic effects of *Bletilla striata* polysaccharide (BSP) on wound healing in diabetes mellitus (DM) and to explore the underlying mechanisms. DM mouse models were induced by high fat-diet feeding combined with low-dose streptozocin injection. To establish diabetic foot ulcer (DFU) models, DM mice were wounded on the dorsal surface. Subsequently, mice were treated with vehicle or BSP for 12 days and wound healing was monitored. The effects of BSP on the production of interleukin-1β (IL-1β), tumor necrosis factor-α, macrophages infiltration, angiogenesis, the activation of nucleotide-binding and oligomerization (NACHT) domain, leucine-rich repeat (LRR), and pyrin domain (PYD)-containing protein 3 (NLRP3) inflammasome, and insulin sensitivity in wound tissues were subsequently evaluated. Separated- and cultured- bone marrow-derived macrophages (BMDMs) and cardiac microvascular endothelial cells (CMECs) were isolated from mice and used to investigate the effects of BSP on cell viability, reactive oxygen species (ROS) generation, NLRP3 inflammasome activation and insulin sensitivity *in vitro* following exposure to high glucose (HG). BSP administration accelerated diabetic wound healing, suppressed macrophage infiltration, promoted angiogenesis, suppressed NLRP3 inflammasome activation, decreased IL-1β secretion, and improved insulin sensitivity in wound tissues in DM mice. *In vitro*, co-treatment with BSP protected against HG-induced ROS generation, NLRP3 inflammasome activation, and IL-1β secretion in BMDMs, and improved cell viability and decreased ROS levels in CMECs. Moreover, in HG exposed BMDMs-CMECs cultures, BSP treatment suppressed NLRP3 inflammasome activation and IL-1β secretion in BMDMs, and improved cell viability and insulin sensitivity in CMECs. Furthermore, treatment with IL-1β almost completely suppressed the beneficial effects of BSP on the NLRP3 inflammasome, IL-1β secretion, and insulin sensitivity in HG-treated BMDMs-CMECs. BSP promotes DFU healing through inhibition of the HG-activated NLRP3 inflammasome.

## Introduction

Diabetes mellitus (DM) afflicts more than 382 million people globally (Basu et al., 2019). Diabetic foot ulcer (DFU) is a major complication of DM which leads to a poor quality of life in diabetic patients. The lifetime risk of patients with DM developing DFU is as high as 25% (Noor et al., 2015). Accumulating evidence indicates that DFU results from persistent inflammation caused by defective tissue repair responses in DM. Of note, the nucleotide-binding and oligomerization (NACHT) domain, leucine-rich repeat (LRR), and pyrin domain (PYD)-containing protein 3 (NLRP3) inflammasome-mediated inflammatory cascade has been suggested to contribute to the deficiencies in angiogenesis and ulcer healing in DM patients (Mirza et al., 2013; Zhang et al., 2017; Liu et al., 2019; Huang et al., 2020).

The NLRP3 inflammasome is a multi-protein complex composed of NLRP3, apoptosis-associated speck-like protein containing a caspase recruitment domain, and caspase-1, which can be activated under metabolic stress by damage-associated molecular patterns including high glucose (HG), palmitate, cholesterol crystals, uric acid, ceramide and C-reactive protein (Hotamisligil, 2017). Specifically, under sustained hyperglycemia in DM, HG leads to the overproduction of reactive oxygen species (ROS) leading to thioredoxin (TRX)-interacting protein (TXNIP) to dissociate from TRX, enhancing TXNIP expression and activation of the NLRP3 inflammasome (Cha-Molstad et al., 2009; Zhou et al., 2010). Once the NLRP3 inflammasome is activated, the autocatalytic activation of caspase-1 leads to the cleavage of inactive pro-interleukin-1β (IL-1β) into its bioactive forms. Compared to non-diabetic patients, the expression of NLRP3, caspase-1 and IL-1β are significantly higher in the wound tissues of DFU patients (Zhang et al., 2017). It is now recognized that the IL-1β-mediated inflammatory response can reduce insulin sensitivity and damage vascular endothelial cells, leading to defective wound healing (Bitto et al., 2014; Zhang et al., 2017; Liu et al., 2019; Huang et al., 2020). Conversely, blocking either the NLRP3 inflammasome or IL-1β-mediated inflammatory reactions can effectively promote DFU healing (Mirza et al., 2013; Bitto et al., 2014; Mirza et al., 2014; Huang et al., 2020).

*Bletilla striata* (Thunb.) Reichb. f. (*Bletilla striata*) belongs to the genus of *Bletilla* (Family Orchidaceae) and is commonly found in the markets of traditional Chinese medicine. In China, historical materials (such as “Ben Cao Feng Yuan” or “Ben Jing”) document that *Bletilla striata* as a folk medicine to stop bleeding, promote tissue regeneration and accelerate wound healing (He et al., 2017). Polysaccharides are considered the main active ingredient of *Bletilla striata* (He et al., 2017). Accumulating evidence suggests that *Bletilla striata* polysaccharide (BSP) exerts multiple activities including anti-inflammatory (Yue et al., 2016), antioxidant (Chen et al., 2020), immunomodulatory (Wang et al., 2019), anti-aging (Zhang et al., 2015), and enhanced wound (Chen et al., 2018) and ulcer healing (Zhang et al., 2019a). Due to these effects, BSP has been proposed as a potential material for use in wound dressing (Ding et al., 2017), hydrogel (Luo et al., 2010), tissue engineering scaffolds (He et al., 2017), and drug delivery vehicles (Wu et al., 2010). Yu *et al.* reported the effectiveness of BSP for the treatment of DFU in type 1 diabetic rats, through its ability to promote fibroblast infiltration and collagen synthesis in wound tissues (Yu et al., 2011). However, the molecular actions of BSP on DFU remain poorly understood.

Recently, we demonstrated that the suppression of HG-induced NLRP3 inflammasome activation in liver-resident macrophages termed Kupffer cells (KCs), improves hepatic insulin sensitivity (Zheng et al., 2020). Others have highlighted how dysregulated NLRP3 inflammasome activation in macrophages leads to an imbalance of inflammatory reactions, impaired insulin sensitivity and angiogenesis, and delayed healing of DFU (Mirza et al., 2014; Boniakowski et al., 2017; Zhang et al., 2017). Thus, targeting the excessive activation of the NLRP3 inflammasome induced by HG under hyperglycemia conditions represent a promising strategy for the treatment of DFU. However, whether HG-induced NLRP3 inflammasome activation in macrophages can be regulated by BSP, and the subsequent activities of BSP that influence DFU healing remain undefined.

In this study, we investigated the therapeutic effects of BSP in DFU mouse models and further explored its underlying mechanisms both *in vivo* and *in vitro*. We found that BSP promotes DFU healing through its inhibition of the HG-activated NLRP3 inflammasome.

## Materials and Methods

### Preparation of *Bletilla striata* Polysaccharide (BSP)

BSP was prepared as previously described (Chen et al., 2014). Briefly, 500 g dry *Bletilla striat*a (Hubei Shengnongbencao Traditional Chinese Medicine Decoction Pieces, Shiyan, China) was powdered and reflux extracted three times in 4,000, 4,000, and 3,000 ml water for 3, 3, and 2 h, respectively. During each of extraction, the solution was separated by centrifugation at 3000 rpm, and the residues were subjected to further extraction steps. For the production of the polysaccharide precipitate, extracts were co-mixed and treated with four-volumes of ethanol at 4°C overnight. Dried precipitates were then re-dissolved in water to a final concentration of 5% (w/v), and four-volumes of ethanol were re-added. Subsequent precipitates were collected and dried using a freezer dryer (LC-12N-50A; LICHEN, Shanghai, China). The resultant lyophilized BSP powder was stored at 4°C.

### Analysis of *Bletilla striata* Polysaccharide

#### Ultraviolet (UV) Spectroscopy

BSP was dissolved in ultrapure water and ultraviolet (UV) spectra scanning was performed using a Tecan Infinite 200 PRO microplate reader (Tecan Group Ltd., Mannedorf, Switzerland) from 230 to 400 nm in quartz 96-well plates (JingAn Biological, Shanghai, China). Adenosine 5′-triphosphate disodium salt hydrate (ATP; Aladdin Reagent, Shanghai, China) and bovine serum albumin (BSA; Sigma-Aldrich, St. Louis, MO, United States), substrates with respective absorption peaks of 260 and 280 nm, were used as positive controls for UV spectroscopy determination.

#### Carbohydrate and Protein Content

The total carbohydrates in BSP were determined using the phenol-sulfuric acid colorimetric method as previously described (Liao et al., 2019). Protein content in BSP was measured *via* BCA protein assays (Thermo Scientific, Rockford, Illinois, United States) according to the manufacturer’s instructions.

#### Molecular Weight Assessments

The molecular weight of BSP was determined by high-performance gel permeation chromatography (HPGPC). Briefly, BSP was dissolved in 0.1 M NaNO_3_ solution of which 20 μl was loaded into a Waters 1525 HPLC system (Milford, MA, United States) equipped with two columns connected in series Waters Ultrahydrogel^™^ linear column (7.8 mm × 300 mm), a 2410 refractive index detector (Waters Corp., Milford, MA, United States). The eluent was 0.1 M NaNO_3_ at a flow rate of 0.8 ml/min. The temperature of the column was maintained at 30°C. Molecular weights were determined relative to dextran standards (dextran T-20000, M_W_ = 2,000,000; dextranT-150, M_W_ = 133,800; dextranT-40, M_W_ = 36,800; dextran T-10, M_W_ = 9,700; dextran T-5, M_W_ = 2,700). All dextran reagents were purchased from Sigma-Aldrich.

#### Monosaccharide Composition

The monosaccharide composition of BSP was determined using the 3-methyl-1-phenyl-5-pyrazolone (PMP)-labeling procedure as previously described (Liao et al., 2019). Briefly, BSP was hydrolyzed with 4 M trifluoroacetic acid at 110°C for 2 h, and subsequently labeled with PMP. PMP-labeled monosaccharides were identified on an Agilent 1100 HPLC system equipped with a ZORBAX Eclipse XDB-C18 column (4.6 mm × 250 mm), and a G1315B DAD detector (Agilent, Waldbronn, Germany) at a wavelength of 245 nm. The mobile phase was A, acetonitrile and B, 0.1 M phosphate buffer (pH = 6.7) at a flow rate of 0.8 ml/min and a column temperature of 30°C. Mannose, glucosamine, ribose, rhamnose, glucuronic acid, galacturonic acid, galactosamine, glucose, galactose, xylose, arabinose, and fucose were used as standard. The molar ratios of the monosaccharides of BSP were measured on a Dionex ICS-3000 ion chromatography (IC) system with a CarboPac-PA20 column (3 mm × 15 mm), and integrated pulsed amperometry detector (Sunnyvale, CA, United States). The gradient elution conditions were as follows: 0–20 min, 99.2% H_2_O (A), 0.8% 0.25 M NaOH (B); 21.1 min, 94.2% A, 0.8% B, 5% 1 M NaAc (C); 30.0 min, 79.2% A, 0.8% B, 20% C; 30.1–50.0 min, 20% A, 80% B. The flow rate was 0.5 ml/min and the injection volume was 20 μl.

#### Fourier Transform Infrared Spectroscopy (FT-IR) Spectroscopy

The Fourier transform infrared spectroscopy (FT-IR) spectra of BSP were determined using a FT-IR650 spectrometer (Tianjin Gangdong Sci. & Technol., Tianjin, China). BSP (2 mg) was mixed with potassium bromide (0.2 g) and pressed to a transparent film. Scanning was performed at a frequency range of 400–4,000 cm^−1^.

### Animals

Male C57BL/6 mice (6 weeks old) were purchased from Beijing HFK Bioscience Co., Ltd (Beijing, China) and housed at 22 ± 2°C with 45–75% relative humidity, and a 12 h light-dark cycle. All procedures for animal experiments were approved by the Animal Ethics Committee of Taihe Hospital, Hubei University of Medicine (approval number 2017-493).

### Diabetic Wound Madding and Treatment

After acclimatization for 1 week, mice were fed either a regular diet (RD) or a high-fat diet (HFD, 21% fat, 0.15% cholesterol) for 4 weeks. Diabetic mouse models were induced in HFD mice by a single intraperitoneal injection of 90 mg/kg body weight (BW) streptozotocin (STZ, Sigma Aldrich, St. Louis, Missouri, United States) dissolved in citrate buffer (0.1 M, pH 4.5). RD mice were used as normal controls and received an intraperitoneal injection of citrate buffer. Fasting blood glucose (FBG) levels were measured using glucose monitors (LifeScan, Inc., Milpitas, CA, United States) after 72 h of STZ injection. HFD mice with FBG ≥ 11.1 mM were used as the DM model (Zheng et al., 2012) and for further DFU model development. Briefly, mice were anesthetized by intraperitoneal injection of urethane (Sinopharm Chemical Reagent, Shanghai, China) at a dosage of 1 g/kg BW and the dorsum was shaved. After cleaning with an alcohol swab, two 6 mm excisional wounds were made with a dermal punch and covered with waterproof bandages (Nexcare, 3M Consumer Health Care, Minneapolis, Minnesota, United States) (Mirza et al., 2013). Next, DM mice were randomly assigned into two groups: DM plus vehicle (saline) and DM plus BSP solution (5% w/w, in saline). The BSP solutions were applied on the surface of each wound at a volume of 50 μl. RD mice were assigned as normal controls and received saline treatment (*n* = 6). All mice were treated with vehicle or BSP once daily from the day of wounding (d0) until 12 days later (d12). At the end of day 12, mice were killed under anesthesia using urethane, and blood samples and skin wound tissues were collected. For the assessment of insulin sensitivity in wound tissues, mice were intraperitoneally injected with 0.75 U/kg BW insulin for 10 min before being sacrificed (Zheng et al., 2018).

### FBG (Fasting blood glucose), BW (body weight) and Wounds Area Measurements

The FBG and BW of mice were monitored on days 0, 6 and 12. Digital wound images were captured on days 0, 2, 4, 6, 8, 10, and 12 using a scaleplate and analyzed using ImageJ2x software (Wayne Rasband, National Institutes of Health, United States). Wound healing was expressed as the percentage of the original wound area that had healed as previously described (Liu et al., 2014).

### Biochemical Analyses

Total cholesterol (TC), triglycerides (TG), LDL cholesterol (LDL-C), and HDL cholesterol (HDL-C) levels in the serum were determined using commercial kits (Biosino Bio-Technology and Science Inc., Beijing, China) according to the manufacturer’s instructions. Serum insulin concentrations were determined using enzyme-linked immunosorbent assay (ELISA) kits purchased from Mercodia (Uppsala, Sweden). The levels of tumor necrosis factor-α (TNF-α) and IL-1β in both serum and skin wound tissues were measured using ELISA kits purchased from MultiSciences Biotech (Hangzhou, China). Skin wound tissues (∼20 mg) were homogenized in 0.5 ml of cold-PBS containing protease inhibitor cocktail (Roche, Basel, Switzerland) on ice and centrifuged at 4°C at 14,000 rpm for 15 min. TNF-α and IL-1β concentrations in the supernatants were determined by ELISA and normalized to the total protein concentration in the supernatants measured by BCA assay.

### Histological Analysis of Wound Tissues

Skin wound tissues were cleaned and fixed in 10% neutral formalin buffer for 24 h. Samples were then embedded in paraffin and cut into 5 μm thick sections. For immunohistochemical analysis, sections were stained with CD68 (Proteintech Group, Chicago, Illinois, United States) or CD31 (Abcam, Cambridge, Massachusetts, United States) antibodies (1:50 dilutions). Sections were then washed and stained with DAB (Boster Biotechnology, Wuhan, China) and slides were imaged on an Olympus BX51 microscope system. The ratio of macrophages was calculated as the number of nuclei of CD68-positive cells divided by the total number of nuclei in the section, as previously described (Zheng et al., 2015). Sections were stained with CD31 and used to determine the number of capillaries per wound (Liu et al., 2014).

### Cultures of Bone Marrow-Derived Macrophages (BMDMs) and Cardiac Microvascular Endothelial Cells (CMECs)

BMDMs were isolated from male C57BL/6 mice (6–8 weeks old) as previously described (Manzanero, 2012). Briefly, both the femurs and tibias were dissected from the back legs of mice, and the bones were cut. The bone marrow was then flushed using pre-warmed DMEM containing 10% FBS using a 27-gauge needle. Cells were subsequently cultured in DMEM containing 10% FBS and 20 ng/ml M-CSF (PeproTech, Rocky Hill, New Jersey, United States) at 37 °C, 5% CO_2_ for 5 days. Cells were then harvested and plated at 2 × 10^5^ cells/well in 24-well plates for further studies. CMECs were isolated as previously described (Li et al., 2017) and cultured in DMEM containing 10% and 30 ng/ml ECGS at 37 °C, 5% CO_2_. To mimic hyperglycemia and insulin resistance *in vivo*, cells were exposed to 30 mM glucose plus 100 nM insulin as previously described (Li et al., 2011; Zheng et al., 2020). Cells cultured in medium containing 5.5 mM glucose were used as normal glucose (NG) controls.

### Coculture of BMDMs and CMECs

Coculture experiments were performed based on previous studies (Lee et al., 2015; Zheng et al., 2020). Briefly, CMECs were seeded into transwells (Corning, New York, United States) at density of 4 × 10^5^ cells/well for 12 h prior to coculture with BMDMs in 24-well plates. The medium was replaced with DMEM containing 2% FBS, 20 ng/ml M-CSF and 30 ng/ml ECGS in the presence or absence of HG, NG, BSP or IL-1β (PeproTech, Rocky Hill, New Jersey, United States) as indicated. For the assessment of insulin sensitivity, CMECs were stimulated using 10 nM insulin for 20 min prior to protein extraction.

### Cell Viability Assays

Cell viability assays were performed in treated cells using Cell Counting Kit-8 (Dojindo Laboratories, Kumamoto, Japan) according to the manufacturer’s instructions. Data are presented as the percentage of NG controls.

### Oxidative Stress and IL-1β Assays

Intracellular ROS levels were measured in NG, HG or BSP treated cells using dichloro-dihydro-fluorescein diacetate (DCFH-DA) (Beyotime Institute of Biotechnology, Jiangsu, China). DCFH-DA fluorescence was measured on a Tecan Infinite 200 PRO microplate reader. Released IL-1β in the culture medium was detected using ELISA kits (Mercodia).

### Immunoblot Analysis

Protein samples were extracted from skin wound tissues or cultured cells using RIPA lysis buffer (Beyotime Institute of Biotechnology). Proteins were separated through sodium dodecyl sulfate-polyacrylamide gel electrophoresis and transferred to PVDF membrane. Membranes were probed with primary antibodies against TXNIP (Proteintech, Chicago, Illinois, United States), NLRP3, caspase-1, IL-1β, phosphorylated Akt ser^473^ (p-Akt), Akt, phosphorylated GSK3β ser^9^ (p-GSK3β), GSK3β (Cell Signaling Technology, Beverly, Massachusetts, United States) and β-actin (Abbkine, Redlands, California, United States). Membranes were then washed and labeled with horseradish peroxidase-conjugated goat anti-rabbit or goat anti-mouse secondary antibodies (Abbkine). β-actin and all secondary antibodies were used at a dilution of 1:10,000. All other antibodies were used at a dilution of 1:1,000.

### Statistical Analysis

Data are expressed as the means ± SEM from at least three independent experiments. SPSS 13.0 was used for all statistical analysis. An unpaired Student’s *t*-test was used to compare individual groups. Multiple-group comparisons were performed using a one-way ANOVA with post-hoc testing. *p* < 0.05 were considered statistically significant.

## Results

### Characteristics of *Bletilla striata* Polysaccharide

**FIGURE 1:**
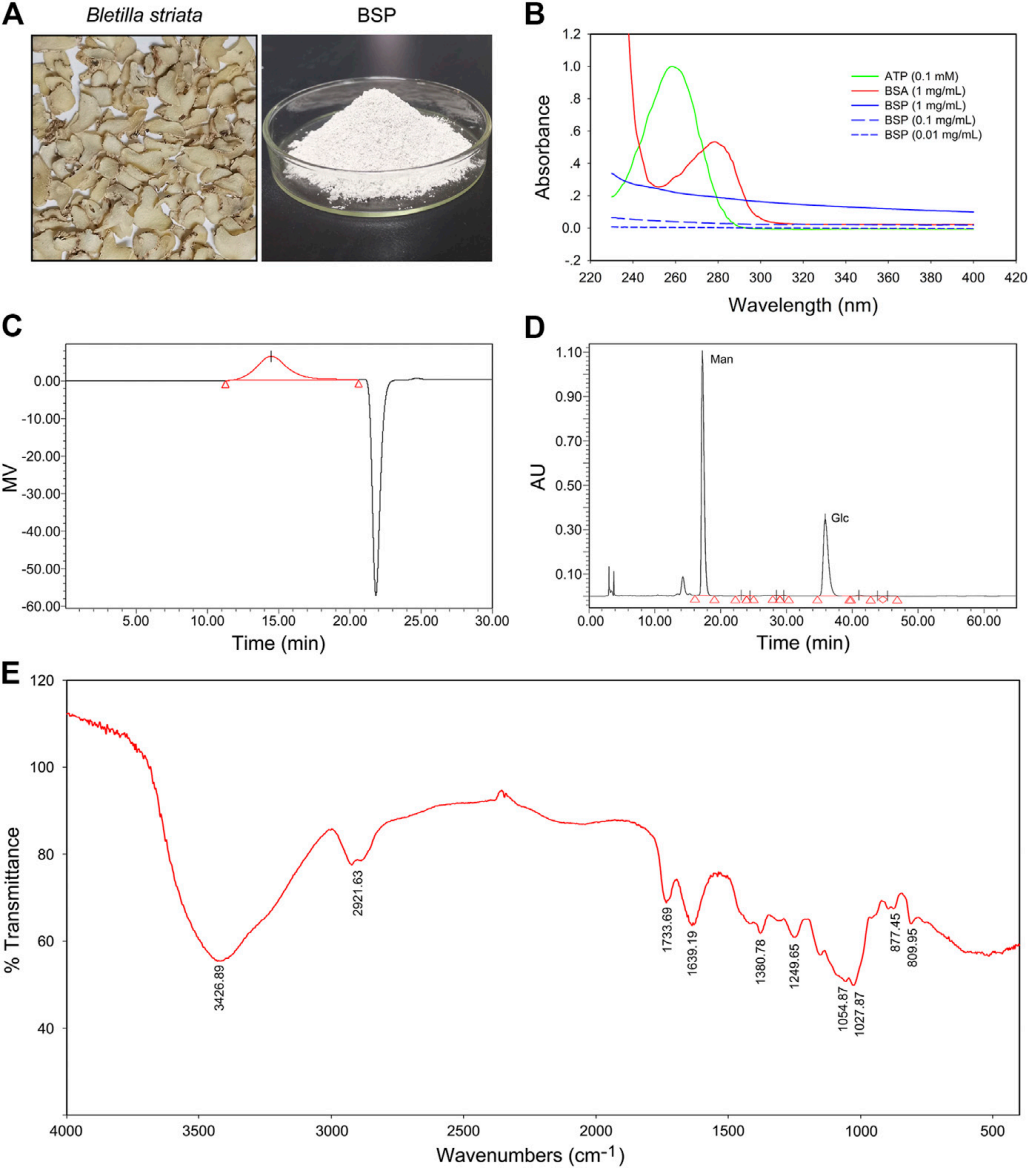
Physicochemical properties of *Bletilla striata* polysaccharide (BSP). **(A)** Images of dry slice of *Bletilla striata* and lyophilized-BSP. **(B)** UV spectra of BSP. **(C)** Elution profile of BSP on HPGPC. **(D)** HPLC chromatogram of derivatives of acid hydrolysates of BSP. **(E)** FT-IR spectra of BSP.

Images of dry slices of *Bletilla striat*a and BSP are shown in Figure 1A. The yield of BSP was approximately 24.5%. UV spectra showed no absorption peaks at either 260 or 280 nm, which contrasted that of ATP or BSA (Figure 1B) confirming that the BSP sample was free of nucleic acids and protein. Carbohydrate content assays revealed a total carbohydrate content of BSP of 99.21%. BCA assays showed no detectable protein in the BSP sample (data not shown), consistent with the UV spectra.

According to HPGPC assays, one of the main polysaccharides was detected in BSP (Figure 1C), with the weight-average molecular weight (Mw) of 536789 g/mol. Others parameters including the number-average molecular weight (Mn), peak molecular weight (Mp), z-average molecular weight (Mz), and the Mw/Mn value of BSP are shown in Supplementary Table S1.

HPLC analysis of PMP-labeled BSP indicated that BSP was observed as a heteropolysaccharide composed of mannose and glucose (Figure 1D), with a relative molar ratio of 1.67:1.00 based on their peak areas. Chromatogram of standard derivatives is shown in Supplementary Figure S1. IC analysis showed that the molar ratio of mannose and glucose was close to 1.84: 1.00.

FT-IR spectra of BSP (Figure 1E) showed an intense and broad band at 3426.89 cm^−1^ that was assigned to O-H stretching vibrations. Bands at 2921.63 cm^−1^ indicated the presence of C-H stretching (Liao et al., 2019). Bands at 1733.69 cm^−1^ and 1639.19 cm^−1^ were assigned to C=O stretching vibration (Bodade et al., 2013; Liao et al., 2019). Bands around 1380.78 cm^−1^ were due to C=O stretching vibrations (Wei et al., 2017). Bands at and 1249.65 cm^−1^ was assigned to O-H or -C-O bending vibrations (Mohanty et al., 2017). The weak signals at 877.45 and 809.95 cm^−1^ were suggestive of the presence of D-mannopyranose and mannose residues (Liao et al., 2019), respectively.

### *Bletilla striata* Polysaccharide Promotes Wound Healing in DM Mice

**FIGURE 2:**
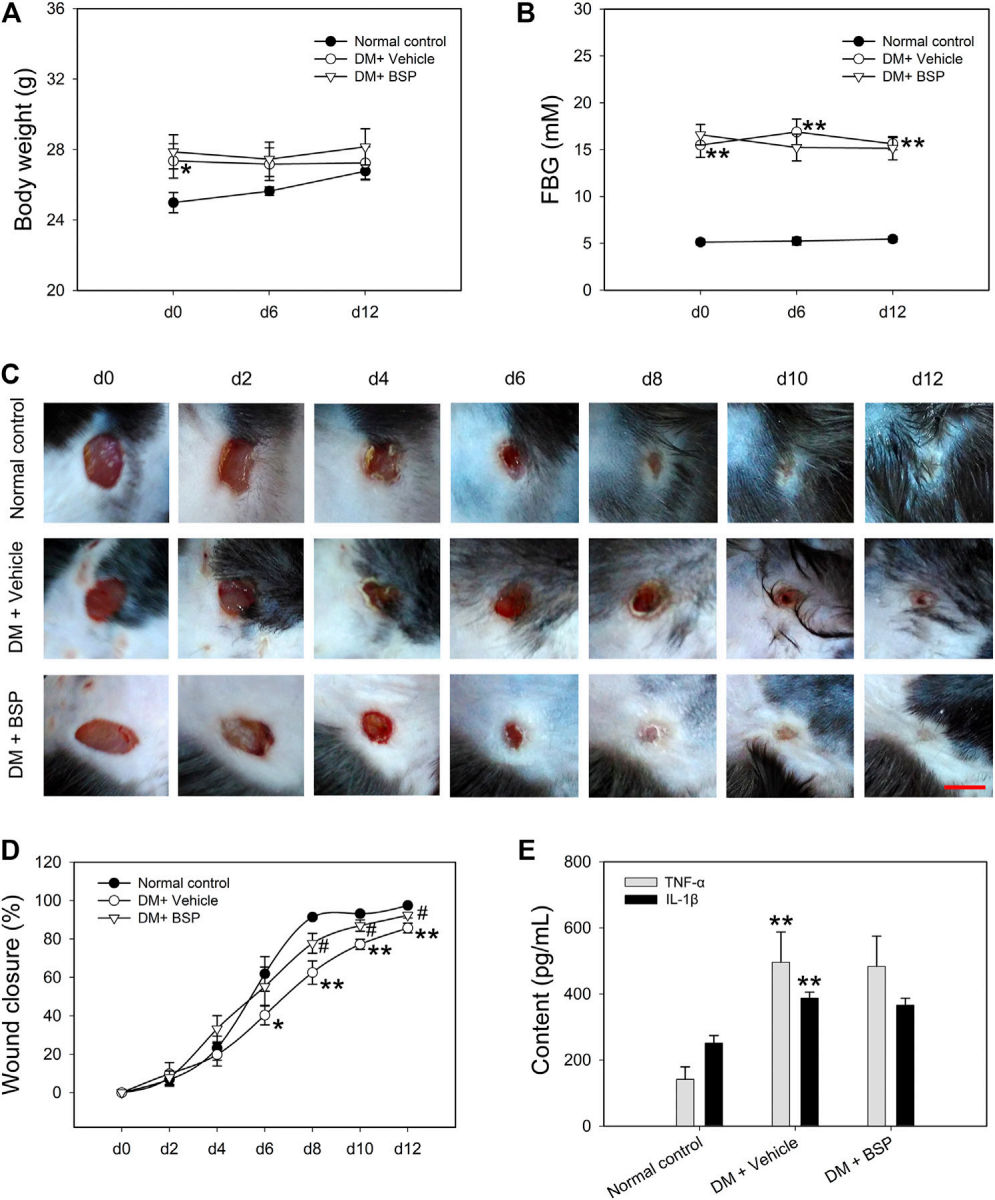
BSP promotes wound healing in DM mice. DM mice received BSP treatment for 12 days. Body weight **(A)** and fasting blood glucose (FBG) **(B)** were measured at day (d) 0, 6, and 12. Wound images of mice grouped in either normal control, DM plus vehicle or DM plus BSP were captured at days 0, 2, 4, 6, 8, 10, and 12 **(C)**. Wound closure was expressed as the percentage of closed wound area **(D)**. Mice were sacrificed and the levels of serum TNF-α and IL-1β were measured **(E)**. Scale bar = 6 mm. *p < 0.05, **p < 0.01 vs. Normal controls; ^#^p < 0.05 vs. DM + Vehicle. Values are means ± SEM (*n* = 6).

The DM model was induced by HFD feeding combined with STZ injection. Compared to RD feeding, HFD mice higher BW at the age of 9 weeks, but no differences in FBG (Supplementary Figures S2A,B). A preliminary experiment to determine the BSP dosage was performed and the results show that BSP concentration-dependently (0.2, 1, and 5%) promoted the wound healing in DM mice (Supplementary Figure S3). In this study, the BSP was used at a concentration of 5% as described in method section. Following drug administration, the BW and FBG of DM mice were higher than normal controls, and BSP treatment did not impact the BW and FBG during the experimental period (Figures 2A,B). Notably, DM plus vehicle mice showed delayed wound healing compared to normal controls. BSP administration effectively accelerated wound closure in DM mice (Figures 2C,D), but did not influence serum TC, TG, LDL-C, HDL-C, or insulin levels (Supplementary Table S2), nor the serum concentrations of TNF-α and IL-1β, which were elevated in DM mice compared to normal controls (Figure 2E). There results confirmed that BSP can promote diabetic wound healing.

### *Bletilla striata* Polysaccharide Decreases CD68 and Increases CD31 Expression in Skin Wound Tissues

**FIGURE 3:**
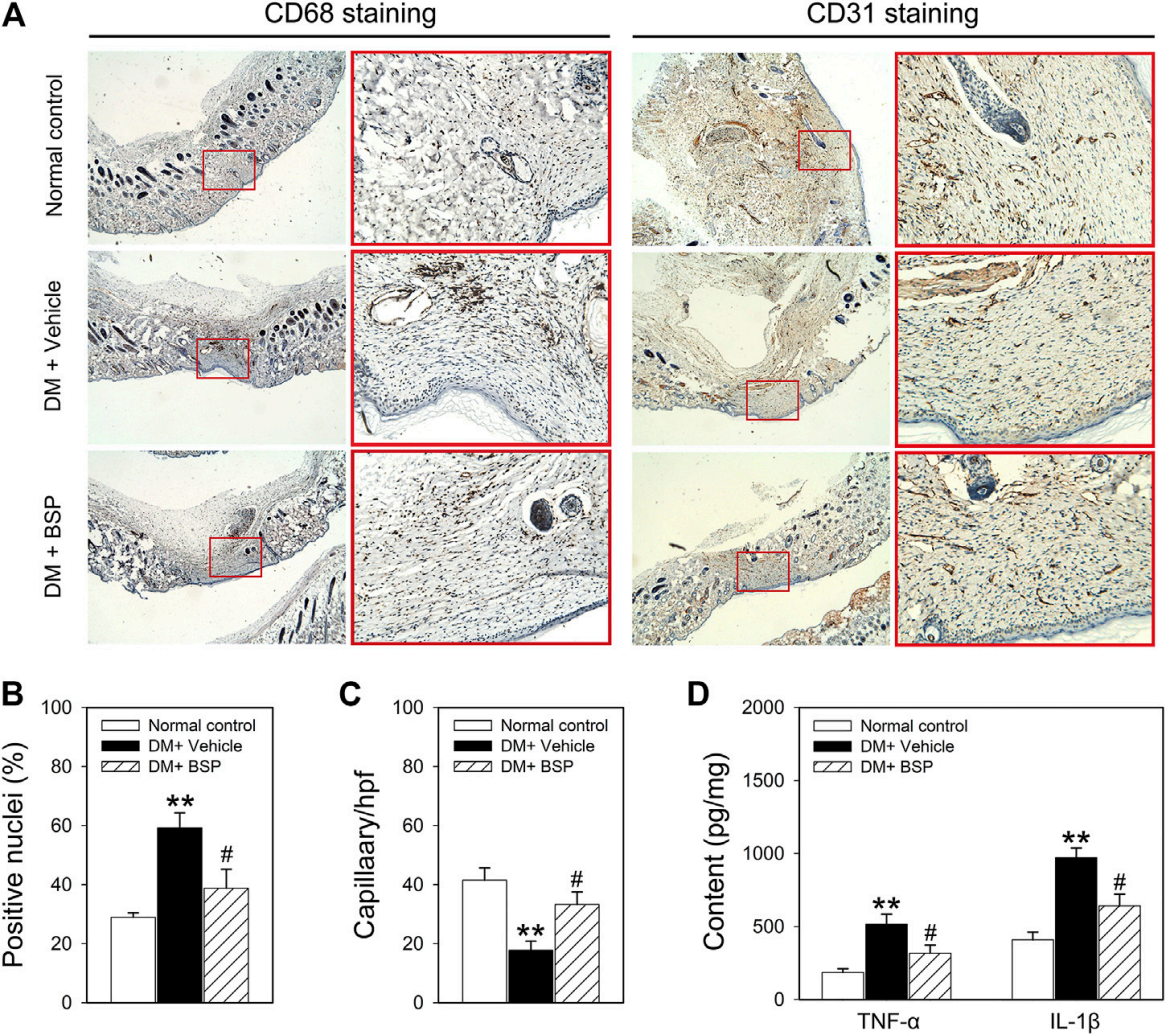
CD68, CD31, TNF-α, and IL-1β expression in the skin wound tissues of mice. After mice were sacrificed, the skin wound tissues of mice were prepared for CD68 and CD31 staining **(A)**, ratios of positive nuclei of CD68 **(B)** and number of capillaries **(C)** determined by stained-CD31 were calculated. Content of TNF-α and IL-1β in skin wound tissues measured by ELISA **(D)**. **p < 0.01 vs. Normal controls; ^#^ *p* < 0.05 vs. DM + Vehicle. Values are means ± SEM (*n* = 3).

CD 68 and CD31 expression were used as markers of macrophage infiltration and angiogenesis in the skin wound tissues, respectively (Liu et al., 2014; Zheng et al., 2015). CD68 and CD31 expression were assessed through immunohistochemical analysis. Figures 3A–C show that compared to normal controls, CD68 was expressed to higher levels in the DM plus vehicle group, which decreased following BSP treatment. Moreover, CD31 staining revealed a lower number of capillaries in the DM model group, which increased following BSP treatment. These results demonstrate that BSP administration improves the imbalances in macrophage infiltration and angiogenesis in the skin wound tissues of DM mice. Dysregulated macrophage infiltration can damage angiogenesis through the local overproduction of inflammatory cytokines such as TNF-α and IL-1β in DM (Mirza et al., 2013; Mirza et al., 2014; Kasiewicz and Whitehead, 2016). We therefore investigated the levels of TNF-α and IL-1β in skin wound tissues. Interestingly, compared to the normal control group, TNF-α and IL-1β content increased in DM mice, and consistent with the results of CD68 staining, decreased in response to BSP treatment in DM mice (Figure 3D). Taken together, these results suggest that BSP improves macrophage infiltration, angiogenesis and TNF-α and IL-1β production in diabetic wounds.

### *Bletilla striata* Polysaccharide Suppresses NLRP3 Inflammasome Activation and Improves Insulin Sensitivity in the Wound Tissues of Mice

**FIGURE 4:**
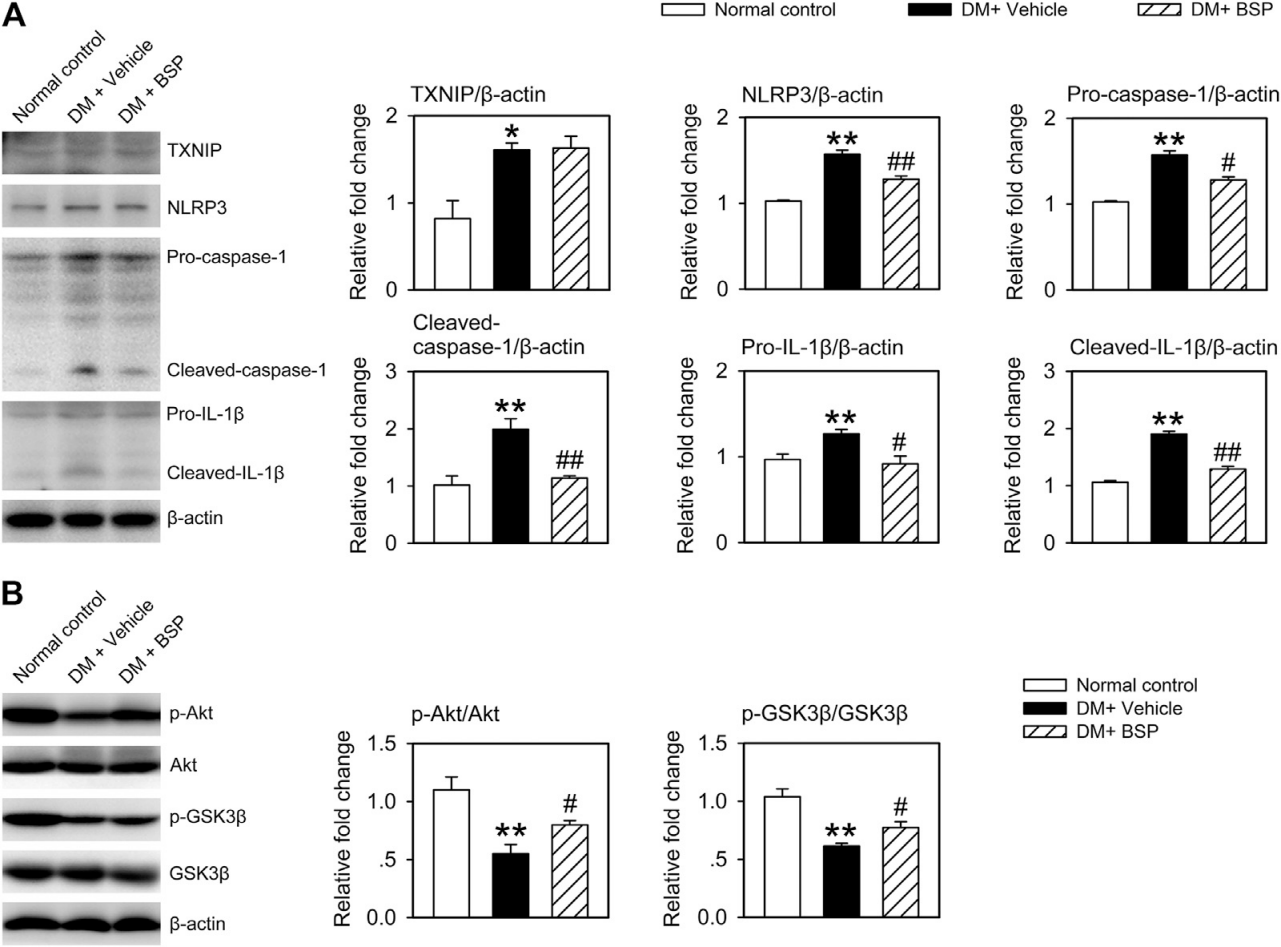
BSP suppresses NLRP3 inflammasome activation and improves insulin sensitivity in skin wound tissues. Mice were sacrificed and the levels of TXNIP, NLRP3, pro-caspase-1, cleaved-caspase-1, pro-IL-1β, and cleaved-IL-1β in lysates from skin wound tissues were determined by immunoblot **(A)**. Levels of phosphorylated Akt and phosphorylated GSK3β in lysates from skin wound tissues in mice receiving insulin injection were determined by immunoblot **(B)**. *p < 0.05, **p < 0.01 vs. Normal controls; ^#^ *p* < 0.05, ^##^ *p* < 0.01 vs. DM + Vehicle. Values are means ± SEM (*n* = 3).

NLPR3 inflammasome activation contributes to the dysregulation of macrophages in DM and delays the onset of wound healing in DFU (Zhang et al., 2017; Huang et al., 2020). We therefore examined whether the NLRP3 inflammasome was suppressed by BSP in the skin wound tissues of DM mice. As shown in Figure 4A, the expression of TXNIP, NLRP3, pro-caspase-1, cleaved-caspase-1, pro-IL-1β, and cleaved-IL-1β in the lysates of skin wound tissues from DM mice increased compared to normal controls. However, DM mice treated with BSP showed decreased levels of NLRP3, pro-caspase-1, cleaved-caspase-1, pro-IL-1β, and cleaved-IL-1β. Additionally, the levels of phosphorylated Akt and GSK3β decreased in DM mice treated with vehicle, but improved following BSP administration, which represented ameliorated insulin sensitivity in skin wound tissues (Figure 4B). Together, these results suggest that BSP suppresses NLRP3 inflammasome activation and improves insulin sensitivity in the wound tissues of DM mice.

### BSP Protects Against HG-Induced Aberrances in Cell Viability, ROS Generation and NLRP3 Inflammasome Activation *In Vitro*

**FIGURE 5:**
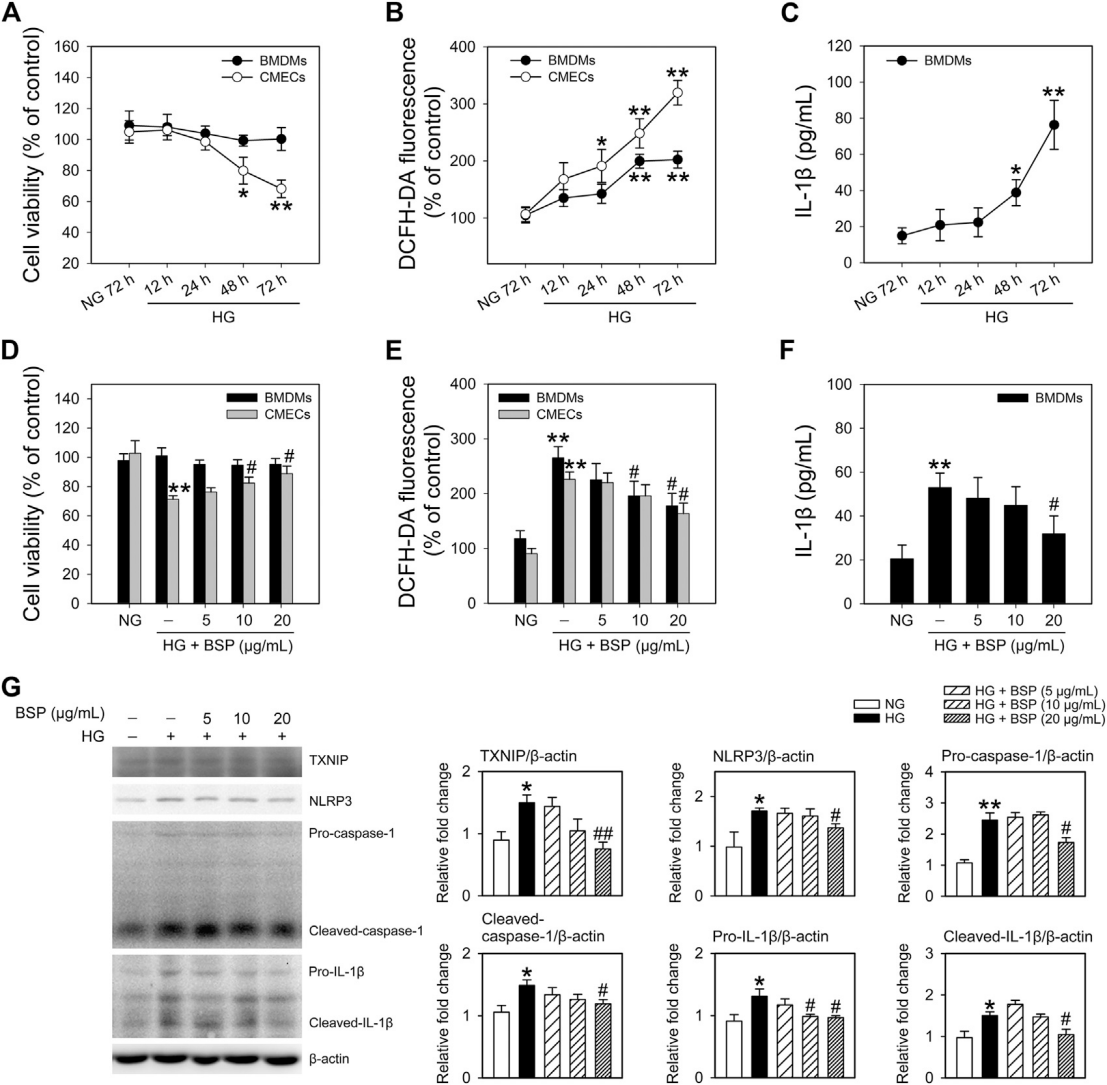
BSP protects against the high glucose-induced decrease in cell viability, ROS generation and NLRP3 inflammasome activation in cultured bone marrow-derived macrophages (BMDMs) and cardiac microvascular endothelial cells (CMECs). After overnight culture in 2% FBS containing normal glucose (NG) at 5.5 mM, BMDMs and CMECs were respectively cultured in 2% FBS containing NG or 30 mM glucose plus 100 nM insulin (HG) for the indicated times (12–72 h). Cell viabilities **(A)** and cellular ROS levels **(B)** were then determined. Levels of IL-1β in the culture supernatant of BMDMs were measured by ELISA **(C)**. After overnight culture in 2% FBS containing NG, BMDMs and CMECs were respectively cultured in 2% FBS containing NG or HG in the presence of the indicated concentrations of BSP (5, 10, and 20 μg/ml) for 48 h. Cell viability **(D)**, cellular ROS levels of BMDMs and CMECs **(E)**, and IL-1β levels in culture supernatants of BMDMs **(F)** were determined as described. Levels of TXNIP, NLRP3, pro-caspase-1, cleaved-caspase-1, pro-IL-1β, and cleaved-IL-1β in cell lysates from BMDMs were determined by immunoblot **(G)**. *p < 0.05, **p < 0.01 vs. NG controls; ^#^ *p* < 0.05, ^##^ *p* < 0.01 vs. HG controls. Values are means ± SEM (**A**, **C**, and **G**, *n* = 3; **B**, **D**, **E**, and **F**, *n* = 4).

To confirm the effects of BSP on the NLRP3 inflammasome, BMDMs and CMECs were cultured in HG media to mimic hyperglycemia and insulin resistance as previously described (Li et al., 2011; Zheng et al., 2020). As shown in Figure 5A, HG treatment (12–72 h) did not impact the viability of BMDMs, but significantly decreased the viability of CMECs after 48 h of HG exposure compared to NG controls. Moreover, HG incubation enhanced ROS generation in BMDMs and CMECs in a time-dependent manner (Figure 5B). We next investigated the IL-1β content in the media. The results demonstrated that the exposure to HG (12–72 h) increased IL-1β secretion in BMDMs (Figure 5C) but not CMEC cultures (data not shown). These findings indicate that under HG conditions, the viability of CMECs decreases, IL-1β secretion from BMDMs increases, and cellular ROS generation is enhanced in both cell types. Moreover, we observed that after co-incubation with BSP, the HG-induced effects on the viability of CMECs (Figure 5D), ROS production in BMDMs and CMECs (Figure 5E), and IL-1β secretion in BMDMs (Figure 5F) improved in a concentration-dependent manner. Furthermore, in BSP-treated BMDMs, the HG-induced expression of TXNIP, NLRP3, pro-caspase-1, cleaved-caspase-1, pro-IL-1β, and cleaved-IL-1β also improved (Figure 5G). Overall, these findings suggest that BSP protects macrophages against HG-induced ROS generation, IL-1β secretion and NLRP3 inflammasome activation, and prevents the HG-induced loss of cell viability and ROS balance in endothelial cells.

### *Bletilla striata* Polysaccharide Suppresses the NLRP3 Inflammasome in BMDMs and Improves Insulin Sensitivity in CMECs Following Coculture and HG Exposure

**FIGURE 6:**
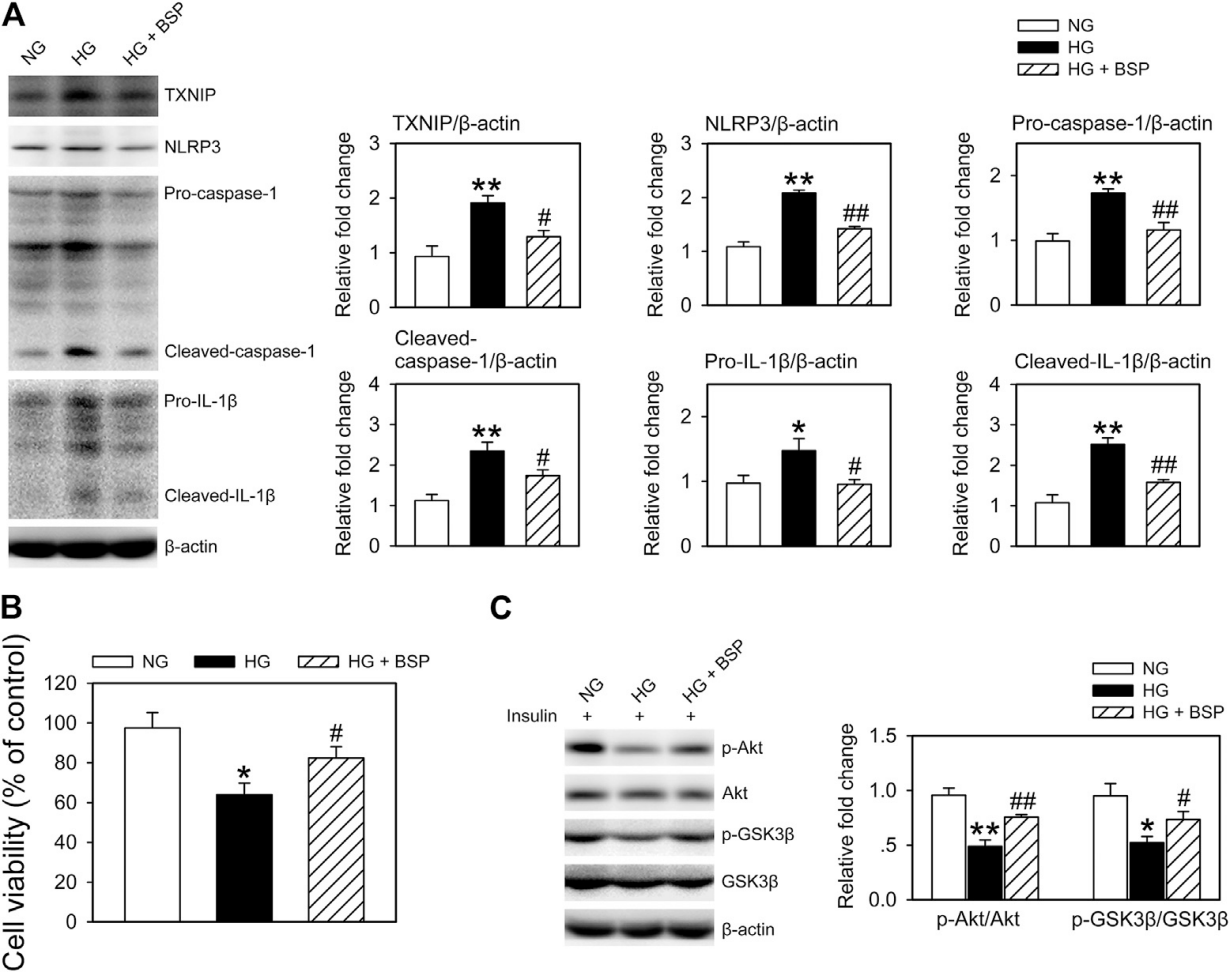
BSP suppresses NLRP3 inflammasome activation in BMDMs and improves insulin sensitivity in BMDMs cocultured-CMECs following HG exposure. Following overnight culture in 2% FBS containing normal glucose (NG) at 5.5 mM, BMDMs and CMECs were cocultured in 2% FBS containing NG or HG in the presence of BSP (20 μg/ml) for 48 h. Subsequently, levels of TXNIP, NLRP3, pro-caspase-1, cleaved-caspase-1, pro-IL-1β, and cleaved-IL-1β in cell lysates from BMDMs were determined by immunoblot **(A)**. Cell viabilities of CMECs were measured as described **(B)**. Cocultures of BMDMs-CMECs were treated as above, and CMECs were stimulated by 10 nM insulin for 20 min alone. Akt and GSK3β phosphorylation were analyzed by immunoblot to assess insulin sensitivity **(C)**. *p < 0.05, **p < 0.01 vs. NG controls; ^#^ *p* < 0.05, ^##^ *p* < 0.01 vs. HG controls. Values are means ± SEM (*n* = 3).

As macrophage-mediated inflammatory cascades contribute the etiology of DFUs (Zhang et al., 2017; Liu et al., 2019; Huang et al., 2020), we cocultured BMDMs with CMECs exposed to HG for 48 h in the presence or absence of BSP. As shown in Supplementary Figure S4, compared to HG exposure alone, the levels of insulin stimulated p-Akt and p-GSK3β in CMECs decreased following they cocultured with BMDMs, indicating that the co-incubation with BMDMs impairs insulin sensitivity in CMECs exposed to HG. Figure 6A shows that BSP effectively suppressed the HG-induced increase in TXNIP, NLRP3, pro-caspase-1, cleaved-caspase-1, pro-IL-1β, and cleaved-IL-1β in BMDMs. In addition, BSP incubation increased the viability of CMECs cocultured with BMDMs exposed to HG (Figure 6B). Importantly, to assess the insulin sensitivity of CEMCs, cells were stimulated by insulin for 20 min prior to lysis. As shown in Figure 6C, cocultured with BMDMs under HG conditions blunted the insulin-stimulated phosphorylation of Akt and GSK3β in CMECs compared to those cultured in NG. However, treatment of BMDMs-CMECs cocultures with BSP significantly increased p-Akt and p-GSK3β levels. Together, these data indicate that BSP not only inhibits HG-induced NLRP3 inflammasome activation in macrophages, but also protects endothelial cells against HG stimulation- and macrophage coculture induced damages on cell viability and insulin sensitivity.

### *Bletilla striata* Polysaccharide Exerts Beneficial Effects on CMECs Through Inhibition of the NLRP3 Inflammasome in Cocultured-BMDMs

**FIGURE 7:**
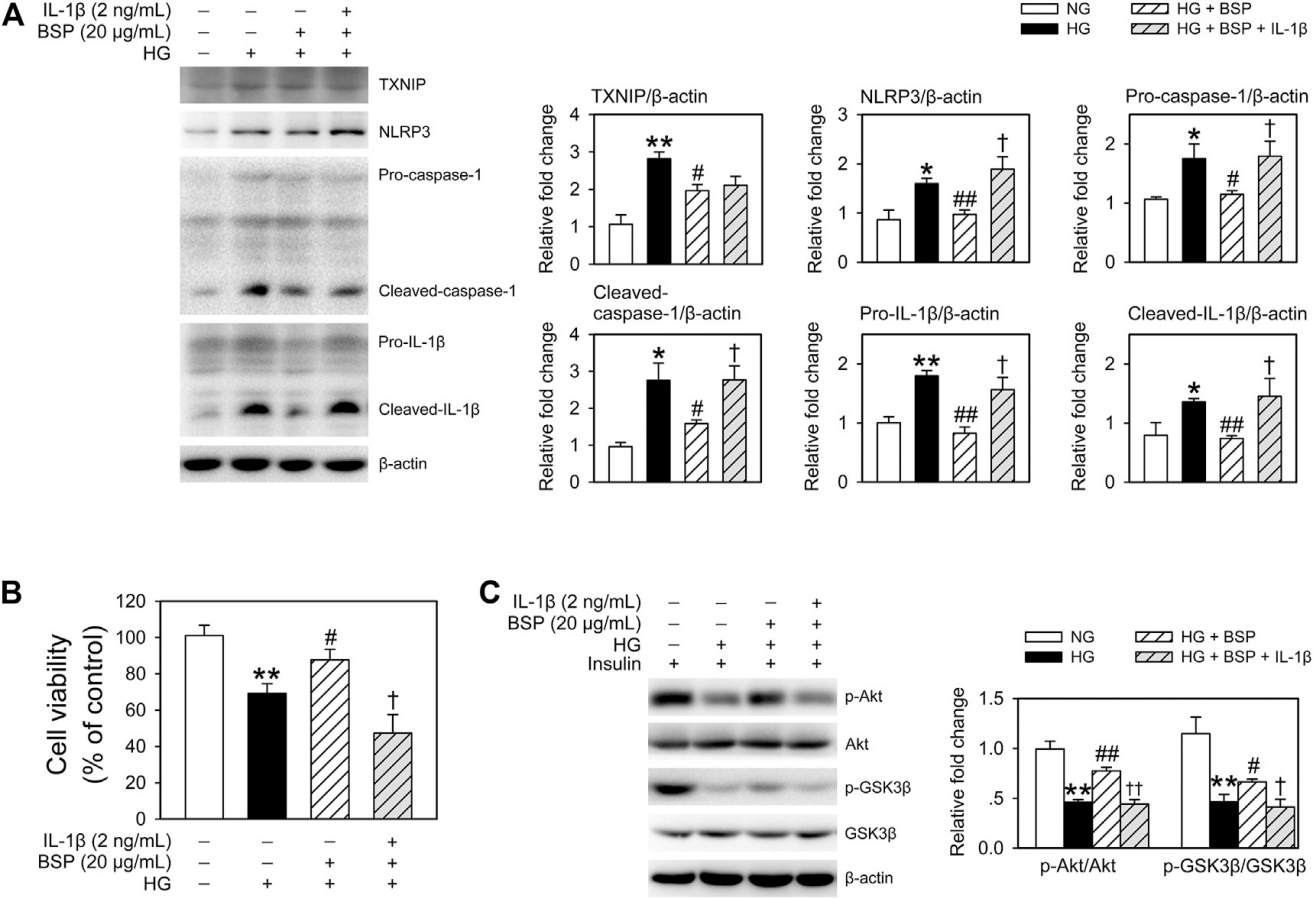
Treated BMDMs-CMECs cocultures with IL-β extinguished the regulatory effects of BSP on the NLRP3 inflammasome in BMDMs, and on cell viability and insulin sensitivity in CMECs. After overnight culture in 2% FBS containing normal glucose (NG) at 5.5 mM, BMDMs and CMECs were cocultured in 2% FBS containing NG or HG in the presence of BSP (20 μg/ml) or IL-1β (2 ng/ml) for 48 h. Levels of TXNIP, NLRP3, pro-caspase-1, cleaved-caspase-1, pro-IL-1β, and cleaved-IL-1β in cell lysates from BMDMs were determined by immunoblot **(A)**. Cell viabilities of CMECs were measured as described **(B)**. BMDMs-CMECs cocultures were treated as above and CMECs were stimulated with 10 nM insulin for 20 min. Akt and GSK3β phosphorylation were analyzed by immunoblot to assess insulin sensitivity **(C)**. *p < 0.05, **p < 0.01 vs. NG controls; ^#^ *p* < 0.05, ^##^ *p* < 0.01 vs. HG controls; ^†^ *p* < 0.05, ^††^ *p* < 0.01 vs. HG + BSP. Values are means ± SEM (*n* = 3).

To confirm the influence of NLRP3 inflammasome activation induced by HG in BMDMs on cell viability and insulin sensitivity of cocultured-CMECs, cocultures were treated with recombinant IL-1β. As shown in Figure 7A, treatment with IL-1β almost completely suppressed the effects of BSP on the expression of NLRP3, pro-caspase-1, cleaved-caspase-1, pro-IL-1β, and cleaved-IL-1β in BMDMs after HG exposure. Moreover, IL-β treatment abolished the protective effects of BSP on cell viability of CMECs after exposed to HG (Figure 7B). Furthermore, IL-β treatment suppressed the effects of BSP on the levels of p-Akt and p-GSK3β in CEMCs (Figure 7C). Taken together, these findings suggest that BSP protects against HG-induced damages on cell viability and insulin sensitivity in endothelial cells, in-part through its ability to inhibit NLRP3 inflammasome activation in HG-treated macrophages.

## Discussion

The present study demonstrated that the therapeutic effects of BSP on DFU are mediated through its suppression of HG-induced NLRP3 inflammasome activation in macrophages, which improves insulin sensitivity in endothelial cells.

DFU represents the most common cause of hospitalization in patients with DM, which not only reduces the quality of life of DM patients, but causes a large burden to healthcare systems and society (Yazdanpanah et al., 2018). There is an urgent need to understand the pathogenesis of DFU and identify effective treatment strategies. Accumulating evidence suggests that chronic inflammation is key to the development of DFU (Dinh et al., 2012; Mirza et al., 2013; Bitto et al., 2014; Noor et al., 2015; Kasiewicz and Whitehead, 2016). Notably, NLRP3 inflammasome activation in response to HG in DM, leads to IL-1β-evoked inflammatory cascades that contribute to the delay in DFU healing (Mirza et al., 2014; Zhang et al., 2017; Liu et al., 2019; Huang et al., 2020). Recent report suggests that the sustained-activation of the NLRP3 inflammasome is a common feature in macrophages in the wounds of diabetic humans and mice, and blocking IL-1β activity using neutralizing antibodies, caspase-1 inhibitors and *Nlrp3* or *caspase-1* gene silencing can effectively improve DFU healing in mice (Mirza et al., 2014). Additionally, the use of BAY11-7082 and Brilliant blue G to inhibit the NLRP3 inflammasome improves healing in DM mice (Bitto et al., 2014). Regulation of the NLRP3 inflammasome therefore represents an effective approach for the treatment of DFU.

Our current findings demonstrate that following BSP treatment, DM mice show significantly accelerated wound healing. In skin wound tissues, BSP treatment corrected the increasing levels of TNF-α and IL-1β, and importantly suppressed the activation of the NLRP3 inflammasome and enhanced the insulin-stimulated levels of p-Akt and p-GSK3β. Evidence from clinical studies suggests that DFU patients with ulcers that fail to heal show higher serum TNF-α levels (Dinh et al., 2012). Our results showed that BSP does not influence the serum levels of TNF-α or IL-1β, but decrease their levels in skin wound tissues. These results indicated that BSP administration affects the local production of TNF-α and IL-1β.

Inflammatory cytokines including IL-1β, TNF-α, and monocyte chemoattractant protein 1 influence macrophages infiltration and angiogenesis in DFU (Dinh et al., 2012; Mirza et al., 2013; Kasiewicz and Whitehead, 2016). Compared to normal controls, macrophage infiltration into the wound area was higher during the proliferative stage (from day 7 to 13) of diabetic wound tissues (Miao et al., 2012). In contrast, silencing TNF-α (Kasiewicz and Whitehead, 2016) or blocking IL-1β activity (Mirza et al., 2013) inhibited macrophage infiltration and induced macrophage switching towards healing-associated phenotypes, subsequently improving DFU healing. In this study, histological analyses indicated that BSP suppressed macrophage infiltration and promoted angiogenesis in skin wound tissues. These findings are consistent with the high levels of TNF-α and IL-1β in skin wound tissues.

In HG treated- BMDMs and CMECs which mimic the macrophages or endothelial cells in skin wound tissues, BSP treatment protected BMDMs against the HG-induced increase in ROS generation, NLRP3 inflammasome activation and IL-1β secretion, and corrected aberrances in cell viability and ROS generation in CMECs. These findings are consistent with the notion that BSP suppresses the activation of the NLRP3 inflammasome and improves insulin sensitivity in skin wound tissues. Accumulating evidence suggests that HG-induced NLRP3 inflammasome activation is mainly dependent on its effects on ROS overproduction that leads to the dissociation of TXNIP from TRX, and the up regulation of *Txnip* gene transcription (Cha-Molstad et al., 2009; Zhou et al., 2010; Zheng et al., 2020). These findings may explain why transplanted diabetic endothelial progenitor cells following MnSOD gene therapy can reduce ROS accumulation and restore wound repair in *db/db* mice (Marrotte et al., 2010). Similarly, blocking ROS with the antioxidants NAC or DPI inhibits NLRP3 inflammasome activation and promotes diabetic wound healing (Mirza et al., 2014). Thus, our data indicated that the ability of BSP to suppress the NLRP3 inflammasome is in-part dependent on its action to decrease ROS overproduction in response to HG exposure.

We recently demonstrated that, compared to hepatocytes cultured alone, cells cocultured with KCs show reduced insulin sensitivity following HG exposure (Zheng et al., 2020). However, silencing NLRP3 in KCs or the depletion of KCs in hyperglycemic mice leads to improved insulin sensitivity in cocultured-hepatocytes and the liver (Zheng et al., 2020). Previous studies suggest that HG exposure enhances the activation of the NLRP3 inflammasome in THP-1-derived macrophages and promotes cells towards inflammatory M1 phenotypic differentiation (Zhang et al., 2017). In pathologic wounds of DM, macrophages with a failure to transition from the inflammatory M1 phenotype to the healing-associated phenotype results in a state of sustained inflammation, a loss of angiogenesis and delayed wound healing (Boniakowski et al., 2017). Our current findings suggest that BSP promotes wound healing through regulation of the macrophage switch towards healing-associated phenotypes, mediated through suppression of the NLRP3 inflammasome. However, further phenotypic assays are required to confirm these findings in further studies.

Furthermore, to mimic intercellular interactions in skin wound tissues, we cocultured BMDMs with CMECs *in vitro*, and showed that BSP treatment suppressed NLRP3 inflammasome activation and IL-1β secretion in BMDMs, and improved the cell viability and insulin sensitivity of CMECs in cocultures of BMDMs-CMECs exposed to HG. However, treatment of BMDMs-CMECs cocultures with IL-1β suppressed the beneficial effects of BSP. There results indicate that the BSP-mediated inhibition of NLRP3 inflammasome activation in macrophages may account for its ability to promote wound healing.

*Bletilla striata* has been long traditionally used to accelerate wound healing in China (He et al., 2017). An increasing number of studies have implicated BSP as the major active ingredient of *Bletilla striata* that promotes either normal- (Zhang et al., 2019b) or diabetic- (Yu et al., 2011) wound healing. Previous studies suggest that BSP improves diabetic wound healing through its ability to influence fibroblast infiltration and collagen synthesis in skin wound tissues (Yu et al., 2011), but the molecular mechanism(s) of this activity remains unclear. Previous *in vitro* studies indicated that BSP promotes endothelial cells growth and enhances the autocrine ability of vascular endothelial growth factor (Wang et al., 2006). BSP was also shown to protect human mesangial cells against angiotensin II-induced ROS generation and proinflammatory cytokine production (Yue et al., 2016). BSP has also been suggested as suitable for medical applications including wound dressings (Ding et al., 2017), hydrogels (Luo et al., 2010), tissue engineering scaffolds (He et al., 2017), and drugs delivery vehicles (Wu et al., 2010). Interestingly, a more recent study demonstrated that *Bletilla striata* oligosaccharides improve the metabolic syndrome through their modulation of the gut microbiota and intestinal metabolites in HFD-fed mice (Hu et al., 2020). These findings highlight the enormous potential of BSP and the need for its assessment for further medicinal exploitation.

In summary, we investigated the therapeutic effects of BSP on DFU, and observed inhibitory activity in the HG-activated NLRP3 inflammasome in macrophages, which promoted a microenvironment with lower levels of inflammation and improved insulin sensitivity in skin wound tissues. We therefore demonstrate that these effects as responsible for the ability of BSP to accelerate wound healing.

## Data Availability

The original contributions presented in the study are included in the article/Supplementary Material, further inquiries can be directed to the corresponding authors.
