# Supplementary material for: Bletilla striata Polysaccharide Promotes Diabetic Wound Healing Through Inhibition of the NLRP3 Inflammasome
Source: Front Pharmacol. 2021 Apr 26;12:659215. doi: 10.3389/fphar.2021.659215 (PMC8110216; doi:10.3389/fphar.2021.659215)
Supplement: Supplementary file 1 [file DataSheet1.docx]

Supplementary Material

Supplementary Table S1. Mn, Mp, Mz and the Mw/Mn value of BSP.

| Mn (g/mol) | Mp (g/mol) | Mz (g/mol) | Mw/Mn |
| --- | --- | --- | --- |
| 75944 | 313324 | 1804356 | 7.20 |


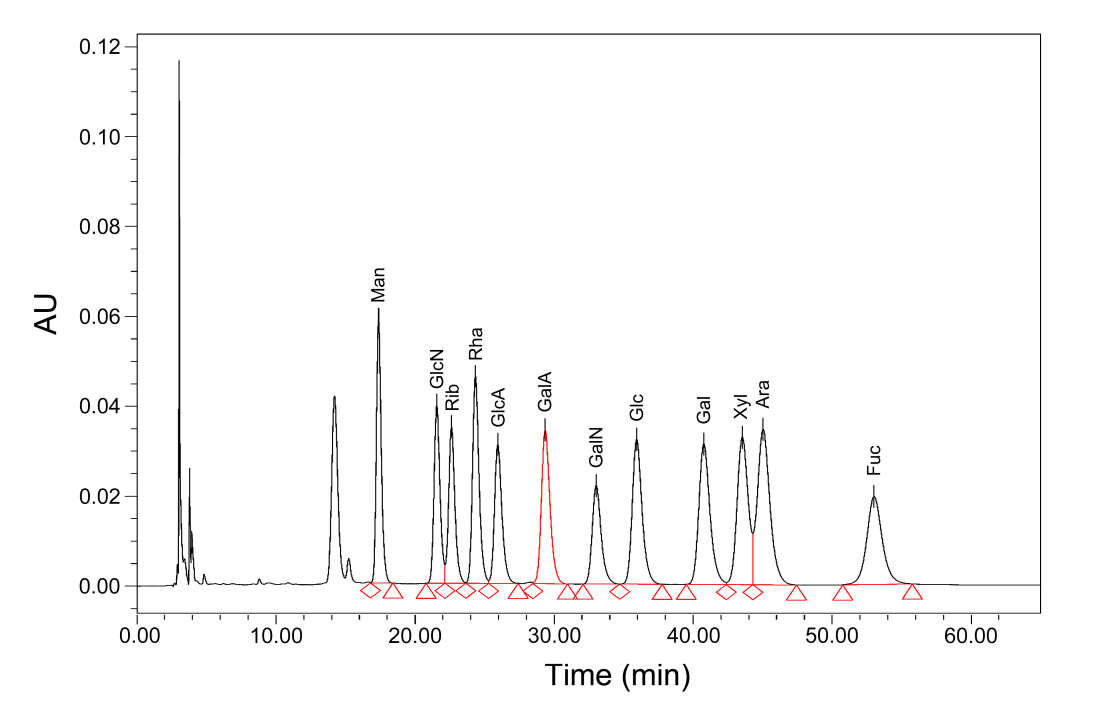


Supplementary Figure S1. Chromatogram of standard derivatives (Man, mannose; GlcN, glucosamine; Rib, ribose; Rha, rhamnose; GlcA, glucuronic acid; GalA, galacturonic acid; GalN, galactosamine; Glc, glucose; Gal, galactose; Xyl, xylose; Ara, arabinose; Fuc, fucose).


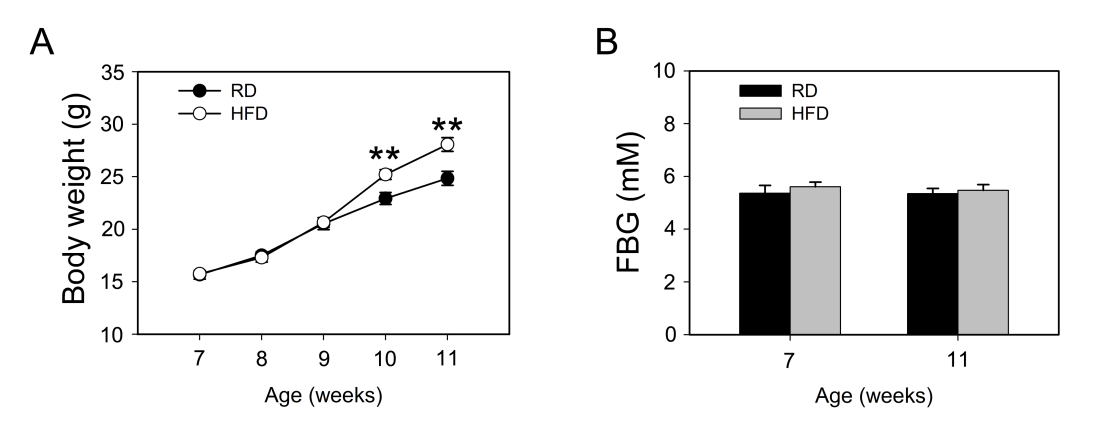


Supplementary Figure S2. Influence of high-fat diet (HFD) on body weight and fasting blood glucose (FBG) in mice. After acclimated, C57BL/6 mice were divided into two groups and they fed with either HFD or regular diet (RD) form age of 7-weeks for 4 weeks. The body weight (A) and the FBG (B) were determined. ** *P* ＜ 0.01 vs. RD. Values are means ± SEM (RD mice, *n* = 10; HFD mice, *n* = 20).


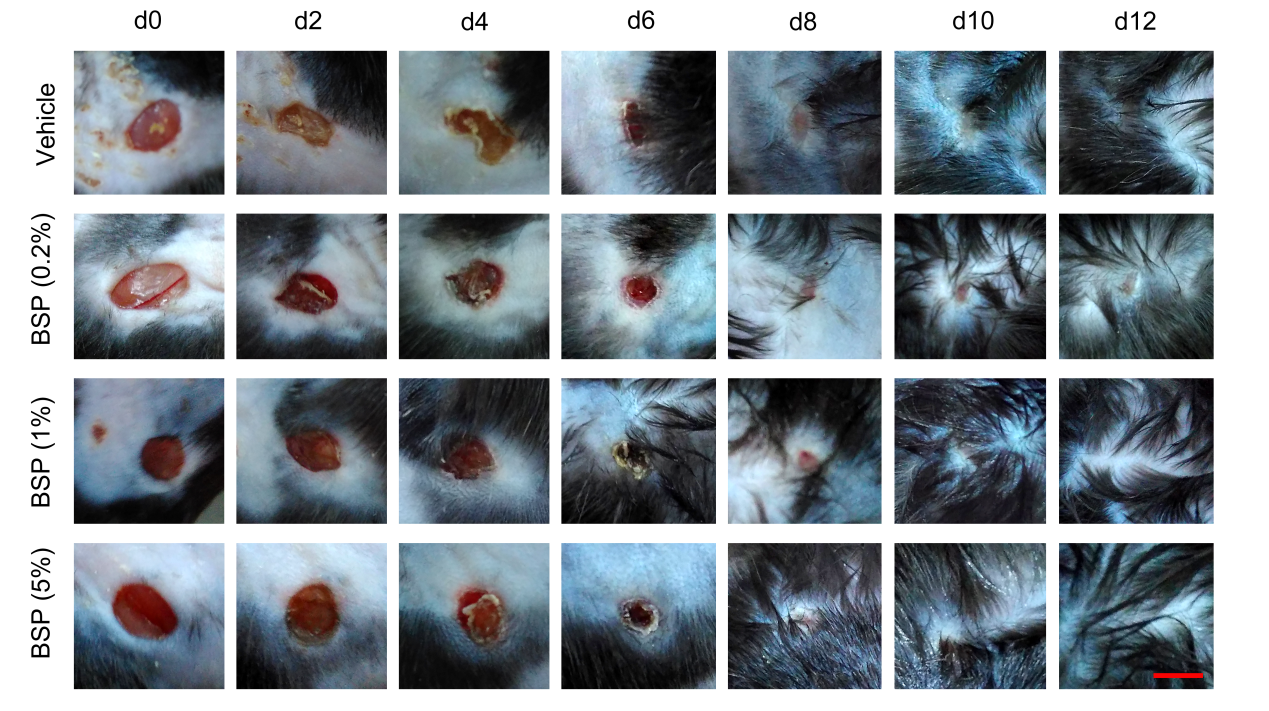


Supplementary Figure S3. Effect of different concentrations of BSP on diabetic wound healing in mice. DM mice received BSP (0.2%, 1% and 5% w/w, in saline) treatment for 12 days. Wound images of mice grouped in DM plus vehicle or DM plus BSP were captured at d0, 2, 4, 6, 8, 10, and 12 (Scale bar = 6 mm).

Supplementary Table S2. The levels of insulin, total cholesterol (TC), triglycerides (TG), low density lipoprotein cholesterol (LDL-C), high density lipoprotein cholesterol (HDL-C), interleukin-1β (IL-1β) and tumor necrosis factor-α (TNF-α) in the serum at the end of the study.

|  | Normal control | DM + Vehicle | DM + BSP |
| --- | --- | --- | --- |
| TC (mg/dl) | 75.38 ± 13.86 | 137.18 ± 28.64* | 144.76 ± 21.99 |
| TG (mg/dl) | 80.86 ± 8.39 | 163.56 ± 19.10** | 129.51 ± 12.22 |
| LDL-C (mg/dl) | 35.55 ± 4.09 | 71.71 ± 12.39* | 68.64 ± 14.59 |
| HDL-C (mg/dl) | 37.75 ± 6.55 | 74.67 ± 15.79* | 80.20 ± 16.91 |
| Insulin (ng/ml) | 1.16 ± 0.30 | 0.46 ± 0.16* | 0.52 ± 0.12 |

* *P*＜ 0.05, ** *P*＜ 0.01 vs. Normal controls. Values are means ± SEM (*n = 6*).


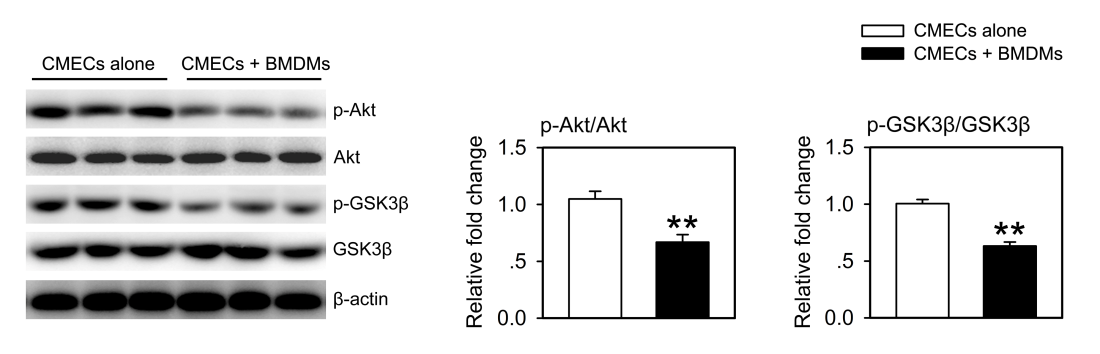


Supplementary Figure S4. Coculturing with BMDMs blunts insulin sensitivity in CMECs after exposure to high glucose. After overnight culture in 2% FBS containing normal glucose at 5.5 mM, CMECs or cocultured BMDMs and CMECs were incubated in 2% FBS containing high glucose for 48 h. Subsequently, CMECs were stimulated with 10 nM insulin for 20 min, and then Akt and GSK3β phosphorylation were analyzed by immunoblot to assess insulin sensitivity. ** *P* ＜ 0.01 vs. CEMCs alone. Values are means ± SEM (*n* = 3).
